# Veterinary Homeopathy in Its Application to the Horse

**Published:** 1897-04

**Authors:** 


### Veterinary Homeopathy in Its Application to the Horse; including a code of common suggestive symptoms. By John Sutcliffe Hurndall, member of the Royal College of Veterinary Surgeons, England. Philadelphia, Boericke & Tafel, 1896. Price, cloth, $2.00 net; by mail, $2.18.

The object the author kept in view in writing this book has been to furnish in concise, and so far as possible, unconventional language information that will enable those who have the charge of horses to discover what ails the animal when signs of illness or incapacity for work are observed, and further how to treat the patient under such conditions. It is an "attempt to cater to those who have no acquaintance either with medicine or disease, but who at the same time are thrown upon their own resources when their equine charges fall ill." It is, therefore, not claimed to be a scientific work, and its language is unconventional and free as possible of scientific terms.

The author has designed the book for "large districts in the United States of America where the services of a qualified veterinary surgeon are not available simply because there is not one resident within a reasonable distance."

In his introduction the author says: "It is quite true that Homeopathy has done what allopathy has failed to do, and it is impossible to assign an intelligible reason for the non-acceptance of the principles of Homeopathy among a large proportion of the medical and veterinary professions. The real fact, stated in plain, unvarnished language, is, that for some unaccountable reason the word Homeopathy and the name of Hahnemann, its discoverer and founder, are like the proverbial red rag to a bull to members of these professions. The bull, without thought, reason or compunction, on seeing a red rag tosses and does his best to destroy it; this is very much like the action of the medical profession toward Homeopathy. A rational person would naturally conclude that on hearing of what Homeopathy could do and had done, the medical profession, whose first duty it is to save life and ameliorate suffering, would hail with joy and thanksgiving any discovery that promised well to realize so desirable an object; and that without loss of time it would fearlessly and honestly investigate the facts and prove them for itself. It is acknowledged that by no means an inconsiderable number has adopted this course, with the result that so far as is known publicly not one single member of the allopathic profession ever undertook the serious investigation of Homeopathy with a conscientious determination to learn the truth, the whole truth, and nothing but the truth, who did not accept the principle as his guide in the future."

There is a chapter on Sanitation and Hygiene, the object of which is "merely to touch upon the main points affecting the sanitary conditions which have a baneful influence on horses of all classes and not to attempt to deal with the subject in extenso."

There is an excellent chapter on Diagnosis, or How to Find Out the Best Course of Treatment. The next chapter deals with the strength of drugs and the dose, potencies, etc., and then come the diseases proper.

In the treatment of Glanders and Farcy, after giving an admirable description of the symptoms so as to enable any horseman to recognize it, the author, in addition to other remedies, recommends Swan's Glanderinum in the CM potency. For Anthrax he recommends the CM potency of Anthracinum and the twelfth potency of Lachesis, as seems to be indicated. In the treatment of Rheumatism we notice he advises

Aconite, Bryonia, Rhus-tox., Belladonna, Caulophyllum, Rhuta-graveolens in the third and sixth potencies; of course giving the indications for each one. For Pyemia he gives Lachesis or Crotalus and mentions the famous case of Dr. Dunham, who was saved from death by pyemia through the administration of Lachesis. Rabies he treats with Belladonna, Hydrophobinum, Stramonium, and Hyoscyamus. Nasal gleet he would treat with Hydrastis in tincture, Kali-bichromicum 3x and Aurum-muriaticum 3x.

These examples suffice to show the value of the book, not alone to the unprofessional man who owns or cares for horses, but to physicians as well. The physician, of course, can very considerably amplify the treatment, while for the layman it is all sufficient.

